# 

**Published:** 1890-12

**Authors:** 


CONTENTS:

PAGE

ORIGINAL ARTICLES:

Etiology of Two Outbreaks of Disease Among Hogs. By John Amory Jeffries, M.D . . . . 681

Malarial Fever in Horses. By Gerald Griffin, D.V.S . . . . 696

Age of the Horse, Ox, Dog, and other Domesticated Animals. By R. S. Huidekoper, M.D., Veterinarian . . . . 702

Open Letter—Texas Fever. By Paul Paquin . . . . 710

Osteo-Porosis. By Dr. George H. Berns . . . . 714

Bubonocele in Horse. By S. D. Brimhall, V M.D. . . . . 719

EDITORIAL:

Dr. Koch's Treatment for Tuberculosis . . . . 720

United States Veterinary Medical Association . . . . 723

SOCIETY PROCEEDINGS:

Iowa State Veterinary Medical Association . . . . 724

Kansas Veterinary Medical Association . . . . 726

Massachusetts Veterinary Association . . . . 726

Long Island Veterinary Society . . . . 726

The Ontario Veterinary College, Toronto, Canada . . . . 727

The Keystone Veterinary Medical Association . . . . 727

REVIEW DEPARTMENT . . . . 730

BOOKS AND PAMPHLETS RECEIVED . . . . 730

